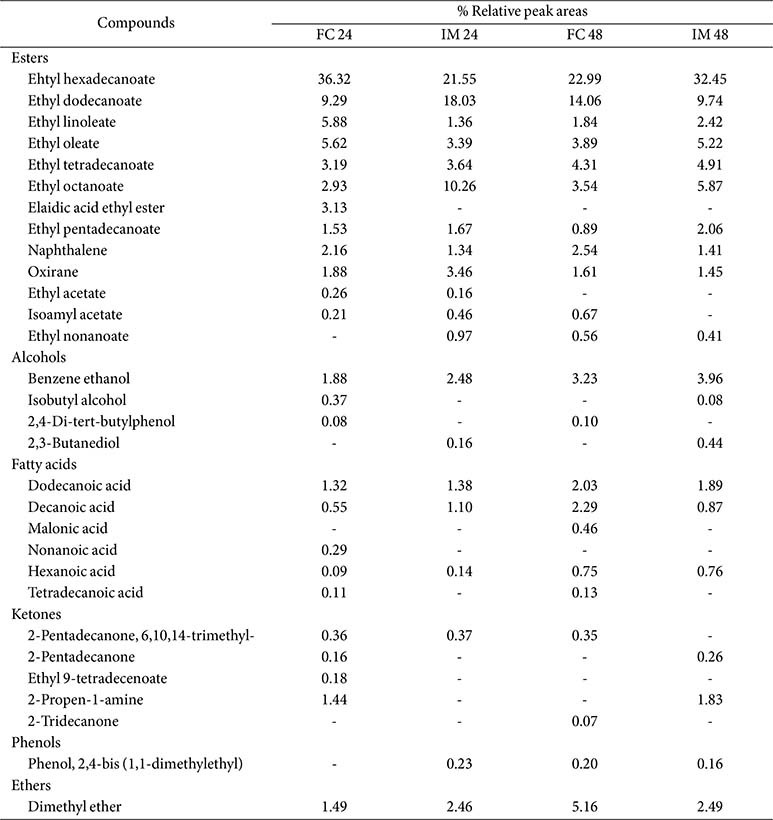# Fruit Rind Peel as Promising Support Materials for Immobilizing Yeast Cells and Application in Repeated-Batch Cider and Wine Fermentation with Improved Aromatic Compound Profiles

**DOI:** 10.4014/jmb.2510.10002

**Published:** 2025-12-09

**Authors:** Rawitsara Intasit, Apisara Iadcharoen, Benjamas Cheirsilp

**Affiliations:** Center of Excellence in Innovative Biotechnology for Sustainable Utilization of Bioresources, Faculty of Agro-Industry, Prince of Songkla University, Hat Yai, Songkhla 90110, Thailand

**Keywords:** Fermentation, immobilization, melon wine, repeated batch, volatile components

## Abstract

This study aimed to valorize unqualified, unsold, and too mature melon fruit through cider and wine fermentation. *Saccharomyces cerevisiae* was immobilized on melon rind peel waste and used for repeated-batch fermentation. The immobilized yeast cells (IM-YC) at 10% loading produced alcohol higher than those at 30% and 50% loadings. The fermentation cycle time of 24 h gave an alcohol concentration of 5.0–5.5%, which could be categorized as cider. With longer fermentation cycle time of 48 h, yeast cells produced more alcohol, up to 12.50 ± 0.71%, which was categorized as wine. Volatile component analysis through GC-MS showed that the fermentations using IM-YC on rind peel had stronger fruit aroma and distinct characteristics when compared to those using free cells. These strategies not only help reduce the inoculum preparation cost but also increase the efficiency and aromatic profiles of cider and wine products, and may greatly contribute to the sustainable food industry.

## Introduction

Cider and wine are alcoholic beverages typically made of fruit juice fermented by yeast, often *Saccharomyces cerevisiae* [1-3]. Cider and wine fermentation processes can be used for valorizing unqualified, unsold, and too mature fruit [4, 5]. Melons are horticultural crops that belong to the Cucurbitaceae family and are produced extensively in temperate, subtropical, and tropical regions throughout the world. Melon pulp and rind are primarily colored by carotenoids and chlorophylls. Ascorbic acid, carotenoids, and phenolic compounds are the main antioxidants found in melon; melon fruits also contain carbohydrates and protein. Furthermore, melons eaten as vegetables include bitter components, alkaloids, and flavonoids, all of which enhance the health benefits [6]. In Thailand, melons are grown mainly in the central, northern, and eastern regions, with a total yield area of 168 hectares in the country [7]. Currently, most Thai farmers turn to plant more melon because of its high price, ease of planting, and high fruit yield. This situation leads to more melon supply than market demand.

Wine fermentation using immobilized yeast cells is a rapidly expanding research area due to its beneficial, technical, and economic advantages compared to the conventional free-cell system. These include a shorter lag phase, more rapid and efficient sugar utilization, and higher productivity compared to free-cell fermentation [8]. Nevertheless, the effective manufacturing of alcoholic beverages also depends on developing the fermentation process. The batch fermentation process is typically used in many wine factories. However, there are several disadvantages, especially if the microbes are slow-growing or highly susceptible to product inhibition [9]. On the other hand, a repeated-batch fermentation mode, in which a portion of the fermented broth is periodically recovered and the remaining broth is then employed as an inoculum for the subsequent batch, can reduce the inoculum preparation cost and improve long-term productivity [10].

Various supports have been used for cell immobilization and wine fermentation [11, 12]. Among them, food-grade natural supports such as delignified cellulose materials, gluten pellets, and fruit pieces like apples and quinces are safer and more biocompatible compared to the synthetic supports [5, 13]. However, the fruit pieces are mostly used for juice extraction, and the smashed fruit pieces cannot be used as support materials. During the manufacturing process, the inedible sections of the fruit, such as seeds and rind peels, are not used in juice extraction and are regarded as wastes. Assuming the significant reutilization potential of the rind peels, alternatives should be investigated. Bioprocessing may be employed to valorize them as low-cost materials for other purposes, reduce their detrimental effects on the environment, and enhance food availability and sustainability. The present study then aimed to valorize the melon rind peel as a novel supporting material for yeast cell immobilization and use the immobilized yeast cells to produce cider and wine from melon juice. The performance was compared with the use of free yeast cells. The attachment of yeast cells to the melon rind peel was also confirmed using scanning electron microscopy (SEM). Thereafter, the immobilized yeast cells were applied to the repeated-batch fermentation for cider and wine production. The volatile aromatic characteristics of cider and wine products using immobilized and free yeast cells were also evaluated using gas chromatography-mass spectrometry (GC–MS).

## Materials and Methods

### Preparation of Melon Juice

Melon pieces were blended and strained using cloth sheet to obtain melon juice. The sugar content was adjusted to 22 oBrix by adding sucrose. This concentration was the optimum concentration for winemaking [14]. The pH was adjusted to 4.5 using citric acid. The melon juice was sterilized by adding potassium metabisulphite (KMS) at 0.018% (w/v) and left for 24 h before inoculation.

### Preparation of Inoculum and Immobilized Yeast Cells

*S. cerevisiae* K1-V116 (LALVIN, Canada) was 0.05% (w/v) inoculated to diluted melon juice with drinking water at a ratio of 1:2 (v/v). The yeast culture was incubated at room temperature (30 ± 2°C) and shaking speed of 200 rpm for 24 h prior to its use as inoculum. The inoculation density was controlled by using the same amount of yeast powder for inoculation and the same pre-culture conditions, and the turbidity of the yeast suspension was confirmed at 600 nm using a spectrophotometer (Shimadzu, Japan). For the immobilization of yeast cells, 1×1 cm pieces of melon rind peel (10 g) were added to 100 ml of diluted melon juice and incubated at room temperature (30±2°C) under aerobic condition by shaking at 200 rpm for 24 h and 48 h. The immobilized yeast cells on melon rind peel (IM-YC) were harvested and used as inoculum for repeated-batch fermentation.

### Effect of Melon Rind Peel Loading and Fermentation Time

The IM-YC was added at three different ratios of 10%, 30%, and 50% (w/v) during wine fermentation. The fermentation was conducted under static conditions at room temperature (30 ± 2°C) for 48 h. The IM-YC was repeatedly used for the subsequent batch fermentation by replacing all fermented broth with fresh melon juice. For the free cells, 10% (v/v) of the fermented broth was retained as an inoculum for the subsequent batch. The repeated-batch wine fermentation was performed for four cycles. The effect of fermentation cycle time (24 h and 48 h) on repeated-batch fermentation was also evaluated. The alcohol production, residual sugar in terms of total soluble solids, yeast cells in broth, and immobilized yeast cells were determined.

### Analytical Methods

The melon juice was evaluated for total soluble solids (TSS) using a refractometer (RSG-100ATC, ChgImposs, China). Alcohol content was measured with an ebulliometer (Per Vinum J. Salleron Dujardin, Paris). Total acidity was determined by titrating samples with 0.1 N NaOH. The immobilized rind peel was crushed and diluted to quantify yeast cells before and after fermentation using a hemocytometer (Counting Chamber, BOE-14V, Boeco, Germany). The immobilized melon rind peel was also examined with SEM (FEI APREO S; Thermo Scientific, Netherlands) attached to an energy dispersive X-ray spectrometer (EDX; Oxford Instruments Analytical, High Wycombe, England).

Volatile components were examined using GC-MS (Trace GC Ultra-DSQ II, Thermo Fisher Electron, USA) combined with HS-Solid Phase Micro Extraction (SPME) [15]. Specifically, 0.5 ml of sample was mixed with 2.0 ml of deionized water and 1.5 g of sodium chloride in a 15 ml headspace vial. The vial was sealed with a screw cap fitted with a silicon septum and pre-equilibrated in a thermostatic water bath at 60°C with a magnetic stirrer for 15 min prior to sample injection. The chromatographic column used for GC–MS analysis was the HP-5ms (30 m 0.25 mm 0.25 μm, Agilent J&W, USA). Helium served as the carrier gas at a flow rate of 1 ml/min in splitless mode. Both inlet and transfer line temperatures were maintained at 260°C. The temperature program was set as follows: the initial temperature of 60°C was held for 2 min, then increased to 250°C at a rate of 7°C/min, and maintained at 250°C for 30 min. In the data analysis, a preliminary qualitative assessment was conducted by identifying the retention time of each volatile compound based on with the Wiley 10, NIST 14, and NIST 17 libraries. Compound identification was accepted when the match score ≥ 90% and the percentage of relative peak areas was calculated.

All data presented are mean values from triplicate experiments, and statistical analysis was performed using one-way analysis of variance with Duncan's test for post hoc comparison (*P* < 0.05).

## Results and Discussion

### Yeast Cell Immobilization on Melon Rind Peel

**Fig. 1:**
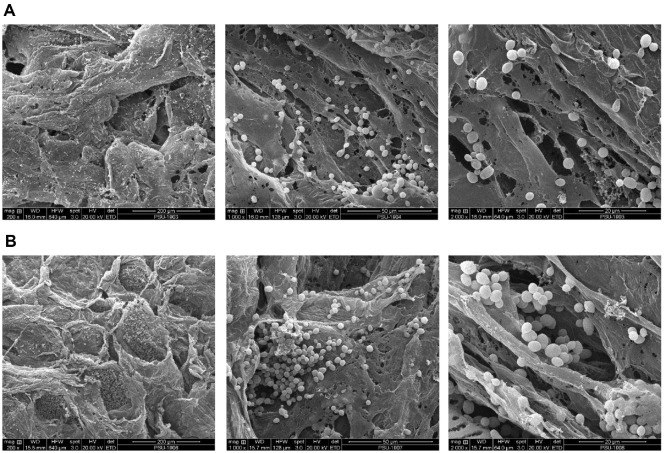
Scanning electron microscopic images of immobilized yeast cells on melon rind peel at 24 h (A) and 48 h (B).

The yeast cells were immobilized on melon rind peels during inoculum preparation. The commercial yeast *S. cerevisiae* K1-V1116 for wine production was inoculated at 0.05% (w/v) in diluted melon juice and incubated at room temperature (30 ± 2°C) and 200 rpm for 24 h and 48 h to immobilize yeast cells. It was found that melon rind peel could immobilize yeast cells up to 7.94 log cells/piece and 8.24 log cells/piece at 24 h and 48 h, respectively. Fig. 1 shows the SEM images of yeast cells immobilized in the porous structure of melon rind peel. Most of them adhered to the internal surface of the melon rind peel. The porous structure and rough surface of the melon rind peel allow yeast cells to adhere and grow intensively. The adhesion would be due to hydrogen bonding, Van der Waals forces, or adsorption by different charges on yeast cell walls and rind peel surfaces [11]. The immobilization for 48 h led to denser yeast cell adhesion compared to the immobilization for 24 h. Among various supports used for cell immobilization, food-grade natural supports, especially inedible sections of the fruit like rind peel, are safer, more biocompatible, and cost-effective compared to the synthetic supports [11-13]. It should also be noted that the use of rind peel waste not only can reduce immobilization costs but also enhance the physicochemical properties of the fermented products and support food availability and sustainability.

Kourkoutas *et al*. [16] immobilized yeast cells on apple, quince, and pear pieces and used them for wine fermentation. They found that immobilized yeast cells on quince pieces gave higher wine productivity than other types of fruit pieces, likely due to the higher amount of immobilized yeast cells. Reddy *et al*. [17], who used guava pieces for the immobilization of yeast cells and applied them to grape wine fermentation, found that the immobilized yeast cells could produce wine with an alcohol concentration up to 12%. Genisheva *et al*. [18], who compared the use of wine-making residues such as grape seeds, skins, stems, and corn cobs (1% (w/v)) as support materials for yeast cell immobilization, found that grape skin and corn cobs could adsorb more yeast cells and gave a higher ethanol production yield of 0.50 g/g. Djordjević *et al*. [8] also found that the immobilized yeast cells exhibited a shorter lag phase, more rapid ethanol production, and more efficient sugar utilization compared to the fermentations using free cells. Fernández-Fernández *et al*. [19] investigated the enhancement of sparkling wine production using immobilized yeast cells. Their findings indicated that the immobilized yeast cells yielded wine with an alcohol concentration of up to 12.85% in the first batch, with a slight decrease to 11.71% in the second batch.

### Effect of Melon Rind Peel Loading on Repeated-Batch Fermentation

**Fig. 2:**
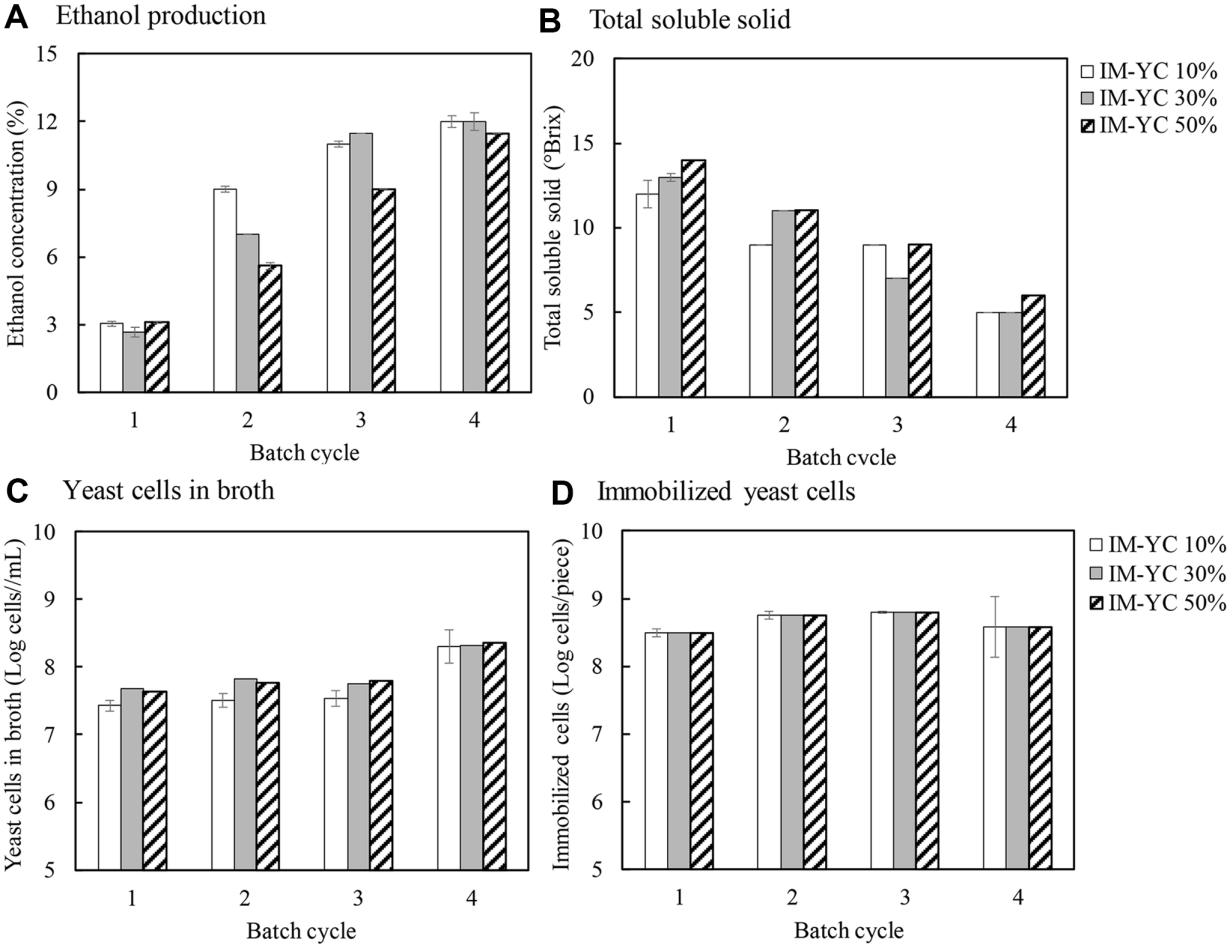
Effect of immobilized yeast cell (IM-YC) loadings on repeated-batch fermentation of melon juice under static condition in flasks for 48 h.

The IM-YC was added at three different ratios of 10%, 30%, and 50% (w/v) during wine fermentation. The fermentation was conducted under static conditions at room temperature (30 ± 2°C) for 48 h. The alcohol production, residual sugar in terms of total soluble solids, yeast cells in broth, and immobilized yeast cells are shown in Fig. 2. The IM-YC was repeatedly used for the subsequent batch fermentation by replacing all fermented broth with fresh melon juice. Since all the fermented broth was replaced with fresh melon juice, the initial ethanol baseline for each batch was very low. The repeated-batch wine fermentation was performed for four cycles. In the first batch of fermentation, there was no significant difference in alcohol production using different IM-YC loadings, while the yeast cells in the broth increased with increasing IM-YC loadings likely due to the larger inoculum and the greater release of yeast cells into the broth. In the second batch fermentation, the IM-YC at 10% loading produced higher alcohol production compared to those at 30% and 50%, despite higher yeast cells detected in the culture broth. It was possible that the high solid loadings might limit mass transfer and alcohol production. The alcohol production increased in the third and fourth cycles, possibly due to more active yeast cells in the subsequent cycles and also because more yeast cells grew in the culture broth. The sugar concentration, which could be observed in terms of total soluble solids, decreased in correspondence to the increase in alcohol production. Nguyen *et al*. [20] studied the immobilized yeast cells in bacterial cellulose (BC) and their use for wine fermentation. They found that the metabolic activities of the immobilized yeast in BC were much higher than those of the free cells. Ton *et al*.[21] also found that the yeast cells immobilized in BC could be repeatedly used for 7 cycles with the maximum alcohol productivity of 1.21 g/l.h. Altieri *et al*. [5] immobilized yeast cells on apple pieces and found that the immobilized yeast cells could be effectively utilized and gave higher ethanol production (6.93 g/l) than the free cells (3.31 g/l).

### Effect of Cycle Time during Repeated-Batch Fermentation

**Fig. 3:**
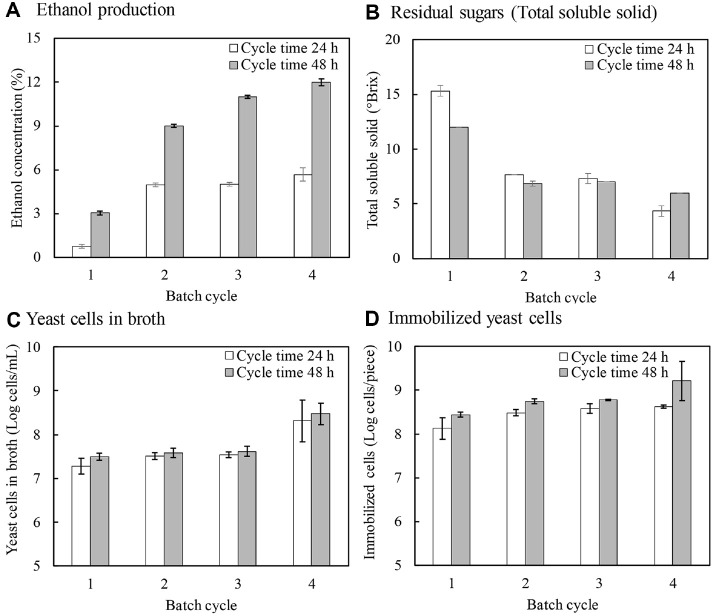
Effect of fermentation time on repeated-batch fermentation under static condition in 5 L jar fermentation by immobilized yeast cells on melon rind peel. The melon rind peel loading was 10% (w/v).

The effect of cycle time during repeated-batch fermentation was evaluated (Fig. 3). It was observed that with a shorter cycle time of 24 h, the yeast cells produced a lower alcohol concentration of 5.0-5.5%, which could be categorized as a cider product. With a longer cycle time of 48 h, the yeast cells produced more alcohol, up to 12.50 ± 0.71% in the fourth cycle. It should be noted that the yeast cells in the broth increased in the fourth cycle, possibly due to the detachment of the growing yeast cells from the melon rind peel. Table 1 shows the physical examination of the winés color and turbidity using a Chroma meter (C.I.E. L*, a*, b*, C*) and C.I.E Lab theory comparison. There was no significant difference between the products fermented for 24 h and 48 h. Both products exhibited high brightness and light transmission in the ranges of 93.42–94.01 and 98.47–98.78, respectively, indicating good clarity and no adverse effects on color due to sedimentation. The yellow color (b*) of both products was in the range of 4.38–5.15, while the red color (a*) was negative for both products, possibly due to the inherent green color of the watermelon flesh, ranging from -0.85 to -0.78. Berbegal *et al*. [22] reported that the immobilized *S. cerevisiae* IOG18-2007 on oak chips and cellulose powder resulted in maximum ethanol production of 12-13%. Altieri *et al*. [5] found that the immobilized yeast cells on apple pieces could be repeatedly used with superior fermentation performance. Liu *et al*. [23] studied the repeated-batch fermentation for greengage wine using free yeast cells and found that the yeast could produce ethanol in repeated batches at 11.27 g/l, 9.90 g/l, and 9.70 g/l, respectively. It should be noted that these values were lower than those of immobilized yeast cells in this study.

### Preliminary Qualitative Analysis of Volatile Constituents

**Fig. 4:**
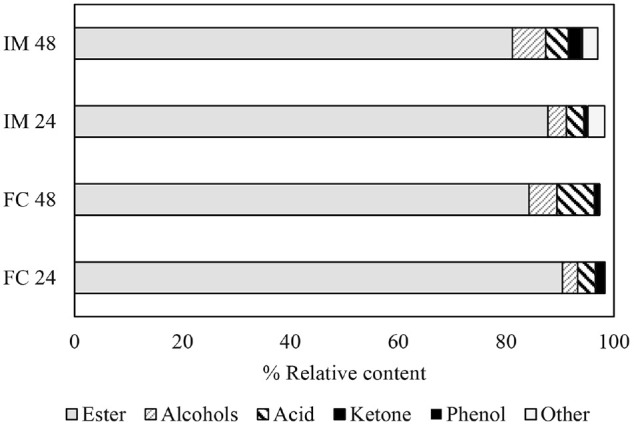
The proportion of main volatile constituents of melon fermentation using free cells (FM) and immobilization cells (IM) for 24 h and 48 h.

Fig. 4 shows the preliminary qualitative analysis of main volatile constituents of melon juice fermentation using free cells and IM-YC for 24 h and 48 h. The volatile compounds were influenced by the use of free cells and IM-YC as well as the fermentation time. Esters are recognized for their beneficial role in enhancing wine aroma. In wine produced by using either free cells or immobilized cells, the ester content at 24 h was found to be higher than that at 48 h. Conversely, alcohols, acids, and ketone contents increased with increasing fermentation time from 24 h to 48 h. Compared with the free cells, the IM-YC exhibited higher alcohol and ketone contents. Using the GC-MS technique, a total of 30 compounds, including esters, organic acids, alcohols, and various other compounds, were detected during melon juice fermentation, as listed in Table 2. Among the ester constituents, ethyl hexadecanoate, ethyl dodecanoate, ethyl linoleate, ethyl oleate, ethyl tetradecanoate, ethyl octanoate, and ethyl acetate, are known to positively influence the aroma of fermented products. Likewise, ethyl octanoate and ethyl decanoate impart a floral, fruity, and slightly musty influence [24]. Ethyl dodecanoate, present in many samples, and ethyl hexadecanoate, detected in wine produced by both free and immobilized yeast cells, are recognized for their aromatic profiles encompassing smoky, earthy, dried fruit, toasty, spicy, candy-like, and herbal notes, respectively [25].

In this study, despite the occurrence of high levels of volatile acidity in certain products, ethyl acetate was detected at a low percentage of relative peak areas. While other studies have indicated that the concentration of ethyl acetate tends to increase in wine produced via immobilized cell techniques [26]. Yeasts can synthesize higher alcohols via direct or indirect pathways during alcoholic fermentation. Among these, straight chain higher alcohols such as benzene ethanol and isobutyl alcohol hold significant quantitative importance due to their robust pungent aroma [27]. Benzene ethanol and 2,3-butanediol, found in some products, are constituents of wine with a mildly bittersweet taste that is unlikely to have significant sensory significance in wine [28]. Analysis of wine produced by IM-YC at 24 h and 48 h revealed a higher level of benzene ethanol compared to the free cells. The wine-making process significantly impacts the formation of higher alcohols during fermentation. Factors such as the presence of oxygen, elevated fermentation temperatures, and the inclusion of skins and suspended solids in the fermenting juice are known to favor their synthesis [24]. It has been reported that these compounds at low concentrations contribute to the aromatic intricacy of the wine [27]. In addition to ethanol, a few fusel and terpene alcohols were identified, albeit in very low concentrations.

Fatty acids were obvious among acids for their potential flavor influence in wines due to their combination of low odor thresholds, relatively high concentrations in wines, and sufficient volatility at ambient temperatures. In this investigation, mainly dodecanoic acid and decanoic acid were detected, known for imparting a rancid, buttery, floral, and cabbage-like aroma [29]. Previous studies have linked higher concentrations of these fatty acids with improved wine quality [30]. Several miscellaneous compounds were also detected, with 1,1-diethoxyethane (acetal) being the most noteworthy. Among the various acetals present in wine products, 1,1-diethoxyethane is likely the only one that could significantly contribute to wine aroma. It has been described as providing a refreshing, fruity, and green odor [30]. Notably, it was detected in the products fermented by both free cells and IM-YC.

According to Nedović *et al*. [31], yeast cell immobilization is a significant technique for facilitating the recycling of biocatalysts and, hence, enhancing fermentation processes that require high cell density and volumetric productivity of target compounds. Additionally, it contributes to the overall enhancement of the sensory properties of final products, such as beer, wine, and cider. Salas-Millán *et al*. [32] observed that the improvement of aromatic compounds in wine products was attributed to an increase in medium-chain fatty acid ethyl esters, such as ethyl hexanoate, ethyl octanoate, and ethyl decanoate, which contributed to a fruity aroma. Liu *et al*. [23] reported that the ester content was significantly elevated, primarily due to increased levels of phenethyl acetate and ethyl benzoate during the repeated-batch fermentation. Lin *et al*. [33] found that the most prominent alcohols found in the apple wine fermentation included 3-methyl-1-butanol, which imparts whiskey, malt, and burnt aromas, and 2-phenylethanol, which contributes honey, spice, rose, and lilac fragrances. The ester profile revealed the presence of ethyl acetate and ethyl isobutyrate, which provide pineapple aromas, resulting in an overall sweet and rubbery scent. Additionally, ethyl decanoate, which produces a grape aroma, was uniquely present in the fermented wine.

## Conclusion

This study has shown that the melon rind peel can be used as a supporting material for anchoring yeast cells and applied in cider and wine fermentation. SEM images revealed the dense adhesion of yeast cells in the porous structure of the melon rind peel. The suitable minimum ratio of melon rind peel to melon juice for alcohol production in the repeated-batch fermentation was 10% (w/v). The fermentation time of 24 h was suitable to produce cider with an alcohol concentration lower than 6%, while the longer fermentation time of 48 h was suitable to produce wine with an alcohol concentration higher than 10%. Wine produced by immobilized cells exhibited high aromatic compounds. It could then be concluded that the fruit rind peels could serve as promising support materials for immobilizing yeast cells and for use in repeated-batch fermentation, which could help reduce inoculum preparation costs and enhance the efficiency and aromatic profiles of cider and wine fermentation.

## Figures and Tables

**Table 1:** Color, turbidity of melon juice fermented by immobilized yeast cells on melon rind peel.

Color	Fermentation time 24 h	Fermentation time 48 h
L*	93.42 ± 0.20^a^	94.01 ± 0.12^a^
a*	-0.78 ± 0.11^a^	-0.85 ± 0.10^a^
b*	4.38 ± 0.10^b^	5.15 ± 0.05^a^
% Turbidity	98.78 ± 0.31^a^	98.47 ± 0.11^a^

Different superscript letters in the same row indicate significant differences between treatments (*P* ≤ 0.05).

**Table 2:** Identification of volatile compounds in the melon fermentation using free cells (FC) and immobilized cells (IM) for 24 h and 48 h.